# Safety assessment of genetically modified plants with deliberately altered composition

**DOI:** 10.1111/pbi.12194

**Published:** 2014-04-16

**Authors:** Nigel G Halford, Elizabeth Hudson, Amy Gimson, Richard Weightman, Peter R Shewry, Steven Tompkins

**Affiliations:** 1 Department of Plant Biology and Crop Science, Rothamsted ResearchHarpenden, Hertfordshire, UK; 2 ADAS UK LtdCambridge, UK; 3 School of Agriculture, Policy and Development, University of ReadingReading, UK

**Keywords:** genetically modified crops, novel traits, crop and food composition, plant biotechnology, risk assessment, food safety

## Abstract

The development and marketing of ‘novel’ genetically modified (GM) crops in which composition has been deliberately altered poses a challenge to the European Union (EU)'s risk assessment processes, which are based on the concept of substantial equivalence with a non-GM comparator. This article gives some examples of these novel GM crops and summarizes the conclusions of a report that was commissioned by the European Food Safety Authority on how the EU's risk assessment processes could be adapted to enable their safety to be assessed.

## Introduction

The food and feed risk assessment strategy for genetically modified (GM) crops in Europe, as applied by the European Food Safety Authority (EFSA) GMO Panel, compares GM plants and their derived food and feed with a conventional non-GM comparator, the comparator being a plant with a history of safe use as food (the principle of substantial equivalence). The development of GM crop varieties that have received substantial, deliberate modifications to their composition, metabolism or physiology raises the question of whether the comparative approach as applied by EFSA would be fully applicable in some cases. Indeed, current EFSA guidance on the risk assessment of food and feed from GM plants states that:

“Where no comparator can be identified, a comparative risk assessment cannot be made and a comprehensive safety and nutritional assessment of the GM plant and derived food and feed itself should be carried out. This would, for instance, be the case where the food and/or feed derived from a GM plant is not closely related to a food and/or a feed with a history of safe use, or where a specific trait or specific traits are introduced with the intention of changing significantly the composition of the plant”

(EFSA, 2011).

Genetically modified varieties with deliberately altered composition are sometimes called ‘second generation’, or described as having ‘output’ traits, distinguishing them from ‘first-generation’ varieties with ‘input’ traits such as herbicide tolerance or insect resistance. The ‘first’ and ‘second generation’ terms are not particularly useful, partly because the first GM varieties to be launched back in 1994 were tomatoes with improved shelf life (an output trait) and partly because GM plants may have output traits combined with well-established input traits. For the purpose of this article, therefore, the term ‘novel traits’ is used. This term also encapsulates the fact that regulatory authorities are much more familiar with GM plants in which the composition of the crop product is indistinguishable from that of its non-GM comparator, such as those carrying herbicide tolerance or insect resistance traits.

### Examples of GM crops with novel traits

Slow-ripening GM tomatoes (reviewed by Grierson, 1996) could be seen as providing a useful precedent for the safety assessment of other GM crop varieties with altered composition. However, they had been developed, launched, marketed and eventually withdrawn years before the current European regulatory system, set out in European Union (EU) directive, GM Food and Feed Regulation (EC) No. 1829/2003, was in place. Calgene's ‘Flavr Savr’ variety, for example, which was marketed in the USA in 1994, was not a commercial success and was soon withdrawn. Tomato paste made from slow-ripening tomatoes was marketed in Europe by Syngenta between 1996 and 1999, but it too was withdrawn at the end of the 1990s.

A more recent example of a GM crop with altered composition and one that has been assessed under the current EFSA risk assessment process is the EH92-527-1 ‘Amflora’ potato variety. ‘Amflora’ was developed by BASF and contained starch composed almost entirely of amylopectin as a result of reduced activity of a granule-bound starch synthase (Visser *et al*., 1991). The aim was to produce starch as an industrial raw material; however, the application for permission to market the variety took 13 years to pass through the EU approval process. It was finally cleared for cultivation in 2010, but BASF announced in 2012 that it was withdrawing from plant biotechnology in Europe altogether, and although ‘Amflora’ is licensed for cultivation, it is currently unavailable.

This outcome highlights the need for robust, workable processes for the safety assessment of GM crops with novel traits. There is some urgency to this because other varieties have either been approved for use elsewhere in the world already, or are believed to be in the final stages of development. One of these is the ‘Mavera’ maize variety produced by Renessen, a joint venture between Cargill and Monsanto, which has been engineered to synthesize more lysine by introducing a gene from a bacterium, *Corynebacterium glutamicum*, encoding a lysine-insensitive dihydrodipicolinate synthase, DHDPS (Huang *et al*., 2005). This variety is aimed at the US bioethanol production market, for which high-protein animal feed is an important co-product. It also contains a ‘triple stack’ of three input traits (two insect resistance and one herbicide tolerance), an example of a novel trait being launched combined with established input traits. Syngenta is also targeting the huge US bioethanol production market by developing a GM maize variety that it claims gives a better yield of ethanol (Johnson *et al*., 2006). The variety contains a *Thermococcales* spp. gene, *AMY797E*, encoding a highly thermostable α-amylase and was deregulated by the US authorities in early 2011.

There are also examples of plant oils being modified by GM. Plant oils contain a range of fatty acids with different chain lengths and degrees of saturation, including some that are polyunsaturated. Polyunsaturated fatty acids (PUFAs) may be considered healthier than saturated fats, but they are prone to oxidation during cooking, giving rise to a rancid, ‘off’ flavour and odour. Food processors can avoid PUFA oxidation by chemical hydrogenation of the double bonds, but this can cause the formation of *trans* fatty acids, which have a similar effect on serum cholesterol levels as saturated fats. Soybean oil is normally rich in linoleic acid, an 18-carbon chain PUFA, and PBI, a subsidiary of DuPont, has produced a genetically modified variety, ‘Plenish’, that accumulates high levels of oleic acid instead of linoleic acid. Hydrogenation, with its risk of *trans* fatty acid formation, is therefore not required for the GM oil.

Other GM plants with more ambitious changes to their oil profile are in development. The longest PUFAs made by higher plants have an 18-carbon chain (C18), but considerably longer fatty acids are present in fish oils, including nutritionally important omega-3 LC-PUFAs such as eicosapentaenoic acid (EPA) (C20) and docosahexaenoic acid (DHA) (C22). EPA and DHA are in fact made by marine algae, not fish, but they accumulate through the marine food chain and are acquired in the human diet from marine fish or farmed fish that have been fed marine fishmeal. The development of GM plants producing EPA and DHA could provide an alternative source of these PUFAs, and the biosynthetic pathway has been successfully engineered into the seed of *Camelina sativa* to produce oil with up to 12% EPA and 14% DHA, levels very close to those in fish oil (Ruiz-Lopez *et al*., 2014).

Increased vitamin content is another obvious nutritional enhancement, with elevated levels of vitamin A in particular being a target in several subsistence crops. Indeed, Golden Rice, a GM line of rice synthesizing the vitamin A precursor, β-carotene, in the grain (unlike its non-GM comparator) was developed in the late 1990s (Potrykus, 2003). Similar technology is being used in cassava and banana, both of which are staple crops in Africa. While these crops are designed to alleviate deficiencies in diets in developing countries, governments may apply for consent to market in Europe in case of inadvertent mixing of the GM varieties with produce destined for export.

Genetic modification is also being used to reduce the level of undesirable substances in food. Asparagine synthetase gene expression in potato, for example, has been reduced by RNA interference to decrease the risk of formation of acrylamide, a probably carcinogenic contaminant that forms from a reaction between free asparagine and reducing sugars during high-temperature cooking and processing (Rommens *et al*., 2008). Other strategies to reduce acrylamide formation target enzymes involved in starch breakdown and in 2013 Simplot Plant Sciences applied to the US authorities for deregulation of low acrylamide GM potato genotypes incorporating one or both of these strategies combined with decreased polyphenol oxidase activity to reduce bruising.

Compositional changes could also result from GM approaches to improve crop tolerance of abiotic stresses, such as drought and salt stress. Drought and salt both impose an osmotic stress and plants use changes in metabolic processes as part of their response, for example by interconverting starch or fructan with simple sugars and synthesizing compatible solutes such as proline and glycine betaine. Strategies aimed at manipulating these responses could result in compositional changes. Lastly, there is biopharming, in which GM plants are used to produce pharmaceuticals, vaccines, antibodies, enzymes or other high-value, nonfood products [reviewed in a previous Commentary (Ma *et al*., 2013)].

### Adapting the EU's risk assessment approaches to cover novel GM traits

The need to adapt the guidance for the risk assessment of GM food and feed to deal with applications for the approval of novel GM traits has been recognized by EFSA, and a report was commissioned in 2013 (Tompkins *et al*., 2013). The report was based on a review of the scientific literature on the topic and the approaches currently being taken by international agencies.

The intention at the start of the review process was to include ‘comprehensive’ risk assessment strategies that did not rely on the comparative approach alone to assess safety. However, it became clear that comparative approaches based on the concept of substantial equivalence (as defined by the OECD in 1993) were the basis of all current risk assessment strategies across the world. In other words, a comprehensive assessment of GM plants and derived food and feed without the use of a comparator is not an approach that is used by international risk assessment bodies or proposed by the scientific literature. Indeed, novel GM plants that have already been approved in the USA and other countries outside the EU have been risk assessed using the same framework as ‘first-generation’ input traits, with no specific modifications to risk assessment criteria, although there are some differences in the way that groups define substantial equivalence in the literature.

A key area of the EFSA risk assessment process that may require revision to make it applicable to novel GM plants is the first step of hazard identification. This is largely because of the difficulty of comparing plants which may have major changes in composition with a conventional comparator. For example, EFSA's guidelines for field trials currently require that a GM variety and its conventional comparator be grown alongside each other to provide material for analysis. This may not be appropriate if, for example, the GM variety has improved stress tolerance because the conventional comparator may not be able to survive in some of the environments in which the GM variety could be expected to be cultivated. In this case, a GM plant may need to be compared with the range of natural variation of control plants grown under ‘normal’ conditions, but with the GM plant grown under the same conditions and in a more extreme environment. A second approach may employ a field experiment to test the response of the GM crop and its comparator to increasing degrees of stress, in the background of a ‘normal’ (i.e. nonstress) environment, in a fully factorial design. The report recommended that further guidance on field trials be developed, including the specific circumstances in which field trial design may need to be amended.

New approaches will also need to be developed to assess novel GM crops containing constituents that are not normally found in that species, with GM oilseeds containing LC-PUFAs that are otherwise found only in fish oils being an obvious example. The concept of ‘history of safe use’, which is already a valuable tool in risk assessment, would be particularly useful here: if an applicant could demonstrate that the novel constituent in the GM plant had a ‘history of safe use’ in food or feed, even if it had previously come from a different source, this would give confidence in its safety. However, while this concept is used by many international risk assessment bodies, it is seldom given a specific definition; in particular, there is no standard requirement for time period or level of consumption. In the report, the definition used by Health Canada was supported, but even this uses vague terms:

“the safety of the expressed trait in food or feed is confirmed from experience of use and continued use in the normal diet of a large part of the population of a country or in farmed/domestic animals for a number of generations, consumed at levels foreseen to be similar to its use in the GM plant”.

It may also be more useful to compare a food product derived from plants carrying the novel trait to another food product that is more similar to it than its conventional counterpart. In the case of GM oils containing LC-PUFAs, this could be fish oil. However, GM plants that have been engineered to synthesize LC-PUFAs do not make true fish oils; they make an oil with a unique combination of some of the fatty acids that would normally be present in that plant species and some additional LC-PUFAs. So, although informative, such a comparison could only be part of the process.

Another consideration would be whether the novel GM crop would change consumption patterns and therefore exposure. This could be difficult to predict because it would be affected by consumer preferences, which could change over time, and the trend by food manufacturers to include functional ingredients in multiple products. It would be particularly important for food constituents for which there is an upper limit to the recommended dietary intake. It would also be important that consumers were given enough information about the difference between the GM and non-GM products to make informed choices over consumption. The results of exposure assessments could be validated by postmarket monitoring (PMM). However, PMM is an area that has received little discussion in the literature, and guidance would have to be developed on the sorts of scenarios where PMM should be performed, what should be measured, the methodologies for carrying it out accurately enough to provide useful data and how it would be financed.

### Concluding remarks

Any system put in place for risk assessing and licensing novel GM traits must work efficiently. This is important not only for the development of these traits (some of which have clear consumer benefits) in Europe, but also because, as the world's most lucrative market for food commodities, Europe's regulatory processes affect the development of crop biotechnology well beyond its borders. The system must also have the confidence of consumers, and the report recommended that public consultation be viewed as an important element of the risk assessment process. In particular, that dossiers of nonconfidential information be published and comments invited before consideration by the GMO Panel and that comments be sought from the public on the panel's initial opinion. This would be similar to the process adopted by the UK's Advisory Committee on Novel Foods and Processes (ACNFP) and in line with the transparency favoured by the United States Department of Agriculture Plant Health Inspection Service. The EU's own Novel Foods Regulations (258/97) are currently being revised, and it will be important to ensure that processes for assessing novel GM traits are aligned with those for assessing other novel foods.
